# Machine Learning Increases Diagnosticity in Psychometric Evaluation of Alexithymia in Fibromyalgia

**DOI:** 10.3389/fmed.2019.00319

**Published:** 2020-01-13

**Authors:** Graziella Orrù, Angelo Gemignani, Rebecca Ciacchini, Laura Bazzichi, Ciro Conversano

**Affiliations:** ^1^Department of Surgical, Medical, Molecular and Critical Area Pathology, University of Pisa, Pisa, Italy; ^2^Rheumatology Unit, Department of Clinical and Experimental Medicine, University of Pisa, Pisa, Italy

**Keywords:** alexithymia, chronic conditions, fibromyalgia, machine learning, experiment

## Abstract

Here, we report an investigation on the accuracy of the Toronto Alexithymia Scale, a measure to assess alexithymia, a multidimensional construct often associate to fibromyalgia. Two groups of participants, patients with fibromyalgia (*n* = 38), healthy controls (*n* = 38) were administered the Toronto Alexithymia Scale and background tests. Machine learning models achieved an overall accuracy higher than 80% in detecting both patients with fibromyalgia and healthy controls. The parameter which alone has demonstrated maximum efficiency in classifying the single subject within the two groups has been the item 3 of the alexithymia scale. The analysis of the most informative features, based on all scales administered, revealed that item 3 and 13 of the alexithymia questionnaire and the visual analog scale scores were the most informative attributes in correctly classifying participants (accuracy above 85%). An additional analyses using only the alexithymia scale subset of items and the visual analog scale scores has shown that the predictors which efficiently classified patients with fibromyalgia and controls were the item 3 and 7 (accuracy = 85.53%). Our findings suggest that machine learning models analysis based on the Toronto Alexithymia Scale subset of items scores accurately distinguish patients with fibromyalgia from healthy controls.

## Introduction

Fibromyalgia syndrome (FMS) has widely been defined as a chronic condition characterized by aching, pain or stiffness in the muscles or joints, and the presence of muscles tenderness (1). The symptoms of fibromyalgia can vary and are similar to those of several other conditions. The literature has suggested that along with the physical symptoms, FMS can be manifest with features belonging to the psychological domains such as depression, anxiety, sleep disorders, fatigue, pain, alexithymia, and functional difficulty of daily living activities (2, 3). In regard to the relationship between depression and FMS, a recent research (4) suggested a shared pathophysiology between the two and demonstrated a bidirectional temporal association which means that the first disease occurring may increase the risk to develop the other. Moreover, a recent study found that pain severity, pain catastrophizing, and fear of movement are influenced by depression and anxiety levels in FMS patients, while fatigue and sleep disturbance may be associated only with depression. Furthermore, both depression and anxiety did not have an amplifying influence on one another when related to somatic and psychological variables (5).

While several psychological domains in FMS patients have been extensively studied, others have been poorly investigated. Among the psychological factors, alexithymia is a personality construct characterized by the “*absence of words for emotion*” which has been minimally explored in FM but is worth examining, as it can negatively impact the perception of emotional sensations. A recent critical review (6), investigating the presence of alexithymia in patients with FMS, highlighted the high prevalence of alexithymia in FM patients. Although the influence of the alexithymic aspect on patients with FMS is not yet clear, it is possible to mention some of the most reliable hypotheses. Alexithymia and FMS share several factors, including a close link with trauma and dissociation as a defense mechanism. In fact, several authors have confirmed the role of traumatic experiences on the onset and the presence of fibromyalgia as well as in individuals with alexithymia (7, 8). Individuals with alexithymia seem to confer much more weight to the physical sensation of arousal instead of interpreting it, which remains suspended somehow. This factor could cause, in FM patients, a considerable distress, the inability to define and integrate emotions, leading to negative consequences regarding social integration and self-acceptance (9).

To date it is possible to affirm that there is no univocal relationship between the two aspects, but that alexithymia leads to an altered interpretation of some emotions and a worsening of the quality of life. Furthermore, a few factors such as pain perception, presence of anxiety, high level of catastrophization, and distress in patients with FMS, should bring the clinicians and health experts back to the importance of the patients' emotional health (10–12). Moreover, alexithymia seems to be associated with an increased affective pain and hypochondriacal illness behavior in FMS patients, with a mediating effect of psychological distress and illness behavior itself (13). Additionally, some researches highlight that not knowing how to reduce the impact of negative feelings—which is typical in individuals with alexithymia—may increase negative affect and chronic sympathetic hyperarousal impairing consequently the immune system (6, 13).

In order to plan and monitor treatments, an increasing number of studies have tried to identify the individuals' features and experience related to the illness across a variety of long-term chronic conditions such as osteoporosis (14), diabetes (15), chronic pain (16), dementia (17), and FMS (18) using comprehensive relevant questionnaires or outcomes measures provided by the medico-clinical research (19).

In the context of FMS, psychological domains that may negatively influence the quality of life of the individuals affected have been poorly investigated, especially in those with a subthreshold clinical manifestation. Due to subthreshold symptoms and/or because of the similarity of symptoms to those of several other conditions, the objective is to identify whether that individual can be accurately identified based on the results of the relevant tests and questionnaires administered. Clarifying the role of specific psychological features in patients with FMS can thus allow better understanding of the disease etiology, the development of patient-tailored treatments, in which both physical and psychological symptoms have to be considered.

Over the past few years, there has been growing interest within the clinical setting in the use of analytical methods in order to efficiently distinguishing patients from healthy individuals on the sole basis of the scores results, enabling to make inferences at both group and individual level. One such method is supervised machine learning (ML), an area of artificial intelligence concerned with the development of algorithms and techniques able to automatically extract information from the available data. In recent years, it has been shown that psychometric testing may be augmented using ML techniques (20) in different fields application, amongst others in neuroimaging (21), malingering (22, 23), genetics (24), and clinical medicine (25). As far as we know, this is the first time that such methods have been applied to fibromyalgia and related psychological domains of interest. ML models have shown promising advantages in solving classification problems. In our case, the issue we intend to address using ML models is the following: Which are the predictors that best distinguish between patients with FMS and healthy controls? In order to guarantee that the reported results are replicable we also evaluated such models using 10-fold cross-validation. Using 10-fold cross-validation the reported results do not suffer from overfitting which yields to overly optimistic results that are not confirmed out of sample. The goal of cross-validation is to test the model's ability to predict new data that was not used in estimating it. The data set is split into 10 folds. In the first iteration, the first fold is used to test the model and the rest are used to train the model. In the second iteration, the second fold is used as the testing set while the rest serve as the training set. This process is repeated until each fold of the 10 folds have been used as the testing set. The average errors an unbiased estimate of the classification accuracy of the model under investigation (26).

## Methods

### Participants

Seventy-six Italian speaking subjects (15 men and 61 women; mean age: 49.71 ± 11.25, range 20–73) were recruited for the present study. The clinical sample consisted of two groups, patients with a diagnosis of FMS (*n* = 38; duration of illness in years: 9.02 ± 6.15, range 1–28), made by an expert rheumatologist, meeting the ACR criteria (27) and healthy controls (HC) (*n* = 38). HC individuals were usually the patients relatives with no history of chronic pain.

The absence of neurological/psychiatric disorders was confirmed for both samples through anamnesis and clinical interview made by a psychiatrist.

The exclusion criteria for FMS and HC group were the following: (1) age ≤ 18, (2) education level below 5 years; (3) poor knowledge of the Italian Language; (4) presence of neurologic or psychiatric disorders.

### Instruments

Socio-demographic data sheet. The data required were the following: age, gender, years of education, marital status. Additional questions about the participants' current mental-health status were asked.

Self-report questionnaire measures, with established norms, were used. All subjects in the FMS and HC group were assessed using the following questionnaires:

Toronto Alexithymia Scale [TAS-20; (28)]. In order to evaluate the presence of alexithymia, a multidimensional construct often associated to FMS (29), the Italian version of the TAS-20 has been used. The TAS-20 individuals items are reported.

Visual Analog Scale [VAS; (30)] and the Italian adaptation of the McGill Pain Questionnaire (31), named “Questionario Italiano sul Dolore” [QUID; (32)] were used to assess the presence, level and type of pain. QUID questionnaire has been administered to evaluate different dimensions of the pain experience: sensory (QUID-S), affective (QUID-A), evaluative (QUID-E), miscellaneous (QUID-M) components and number of words chosen to describe the pain experience (QUID-NWC).

The Italian version of the Hospital Anxiety and Depression Scale [HADS; (33, 34)] was used in order to evaluate symptoms of psychological distress.

### Procedure

FMS patients were recruited at the University of Pisa Hospital (Rheumatology Unit). The HC group was recruited from patients' partners or relatives that accompanied them to visit. The study was approved by Hospital of Pisa Ethics Committee and was conducted in accordance with the Declaration of Helsinki. All the participants gave their written informed consent before to participate in the study.

### Data Analysis

Data analysis has been performed using SPSS and Weka 3.8 (35).

*t*-test has been performed and based on the t values and sample size of the two groups (FMS, *n* = 38; HC, *n* = 38), the effects sizes (d) have been derived through Borenstein's formula (36). The suggestions of Cohen (37) for interpreting the magnitude of effect sizes were the following intervals: 0.0–0.1 (no effect), 0.2–0.4 (small effect), 0.5–0.7 (intermediate effect), 0.8–≥1 (large effect).

In order to evaluate the effectiveness of the TAS-20, the predictors used in developing the machine learning (ML) models were the following: age, education, gender, TAS-20 subset of items, VAS, HADS, and QUID components. The k-fold cross validation technique was used. Specifically, the k = 10 has been used, where the value for k is fixed to 10, a value that has been found through experimentation to have a low bias. In fact, this technique is commonly used in the application of ML techniques in order to compare and select a specific model for a given predictive modeling problem and generally the estimates have a lower bias than other methods.

As a first step we run the classifiers including all the predictors. A second step included a preliminary selection of most informative attributes (38). Such feature selection procedure consists in excluding the predictors which do not contribute to the discrimination between the FMS and the HC groups. We also identified the most discriminative predictor using the OneR algorithm (39). Based on the most informative attributes, the best rule that correctly classify the subject in the two groups was obtained by J48 pruned three algorithm (40).

In order to identify the most efficient items of the TAS-20 scale, we isolated the most efficient subset of items of the same scale through the algorithm developed by Hall (38).

## Results

Means and standard deviations for age, gender, education, and tests scores are reported in Table 1. *t*-test has been performed and based on the t values and sample size of the two groups, the effects sizes (d) have been derived through Borenstein's formula. All questionnaire measures considered have shown effect sizes from small to large according to Cohen's interpretation (37) and are shown in Table 2.

**Table 1:** Demographic characteristics and performance on the administered tests for each group of participants (FMS and HC groups).

	**Group**	***N***	**Mean**	**Std. dev**.
Gender	FMS	38	1.026	0.1622
	HC	38	1.368	0.4889
Age	FMS	38	51.500	12.1160
	HC	38	47.921	10.1512
Education^*^	FMS	38	2.553	0.6857
	HC	38	3.026	0.8849
TAS-20 total score	FMS	38	58.763	13.9040
	HC	38	40.868	10.5937
QUID-S	FMS	38	0.3021	0.12307
	HC	38	0.0955	0.13065
QUID-A	FMS	38	0.3605	0.20966
	HC	38	0.1087	0.16838
QUID-E	FMS	38	0.2903	0.18242
	HC	38	0.0682	0.12096
QUID-M	FMS	38	0.3000	0.23950
	HC	38	0.0584	0.12191
QUID-T	FMS	38	0.3074	0.14993
	HC	38	0.0858	0.11988
QUID-NWC	FMS	38	14.342	6.1039
	HC	38	4.447	5.5298
VAS	FMS	38	6.184	2.3233
	HC	38	2.105	2.4026
HADS total score	FMS	38	19.237	7.5137
	HC	38	7.316	5.1993

TAS-20, Toronto alexithymia scale; QUID components: QUID-S, sensory; QUID-A, affective; QUID-E, evaluative; QUID-M, miscellaneous; QUID-NWC, number of words chosen to describe the pain experience; VAS, visual analog scale; HADS, hospital anxiety and depression scale;

* *Education level: 1 = 5-year; 2 = 8-year; 3 = 13-year; 4 = 17-year*.

**Table 2:** *t*-test and effect sizes (d).

**Variable**	**Student *t*-value**	Effect size (d)^*^
Age	1.396	0.32
Education	−2.608	−0.598
TAS-20 total score	6.311	1.448
QUID-S	7.095	1.628
QUID-A	5.773	1.324
QUID-E	6.255	1.435
QUID-M	5.541	1.271
QUID-T	7.115	1.632
QUID-NWC	7.406	1.699
VAS	7.523	1.726
HADS total score	8.043	1.845

TAS-20, Toronto alexithymia scale; QUID components: QUID-S, sensory; QUID-A, affective; QUID-S, sensory; QUID-A, affective; QUID-E, evaluative; QUID-M, miscellaneous; QUID-NWC, number of words chosen to describe the pain experience; VAS, visual analog scale; HADS, hospital anxiety and depression scale;

* *Effect sizes according to Cohen's (37) intervals: 0.0–0.1 (no effect), 0.2–0.4 (small effect), 0.5–0.7 (intermediate effect), 0.8–≥ 1 (large effect)*.

### Classification Accuracy Between Individuals With Fibromyalgia and Healthy Controls

In applying TAS-20, HADS, VAS, QUID scales in a psychological-clinical setting, the most interesting aspect is to identify which are the main features which enable to characterize patients with fibromyalgia (group A) from healthy controls (group B). In order to pursue this aim, we applied ML algorithms. In the classification process, the parameter which alone has demonstrated maximum efficiency, amongst all domains and subdomains considered, in classifying the single subject within the two groups has been the item three of the TAS-20 (item 3), where the cut-off has been identified through oneR, which yielded an accuracy of 84.21% and the rule was the following:

*if the TAS-20 (item 3) score is* < *3.5, then the subject is classified as a control subject;*

and

*if the TAS-20 (item 3) score is* ≥ *3.5, then the subject is classified as a FMS patient*.

The reported decision rule is not the best classifier, but it gives a better understanding of the principle, which results in high accuracy in classifying FMS (31/38; accuracy = 81%) and HC subjects (33/38; accuracy = 87%), AUC = 0.84 (d = 1.41) and F1 = 0.84.

In Table 3 are displayed the accuracies demonstrated by 5 different classifiers: Naïve Bayes, Logistic Regression, Simple Logistics, Support Vector Machine, Random Forest (using 10-fold cross-validation and 5-fold cross-validation). Classifiers were run with default parameters of Weka and therefore without any parameter tuning.

**Table 3:** Accuracies as measured by % correct, AUC, F1, and correct classification obtained by five different ML classifiers (using 10-fold cross-validation and 5-fold cross-validation).

**Classifier**	**Accuracy (%)**	**AUC**	**F1**	**Correct classification**	
**10-FOLD CROSS-FOLD VALIDATION**					
Naïve Bayes	86.84	0.90 (d = 1.81)	0.87	FMS 34/38	HC 32/38
Logistic regression	72.37	0.78 (d = 1.29)	0.72	FMS 28/38	HC 27/38
Simple logistics	94.74	0.93 (d = 2.09)	0.95	FMS 36/38	HC 36/38
Support vector machine	90.79	0.91 (d = 1.90)	0.91	FMS 34/38	HC 35/38
Random forest	88.16	0.94 (d = 2.20)	0.88	FMS 35/38	HC 32/38
**5-FOLD CROSS-VALIDATION**					
Naïve Bayes	86.84	0.91 (d = 1.89)	0.87	FMS 34/38	HC 32/38
Logistic regression	71.05	0.79 (d = 1.14)	0.71	FMS 29/38	HC 25/38
Simple logistics	86.84	0.87 (d = 1.59)	0.87	FMS 33/38	HC 33/38
Support vector machine	88.16	0.88 (d = 1.66)	0.88	FMS 34/38	HC 33/38
Random forest	86.84	0.95 (d = 2.32)	0.87	FMS 34/38	HC 32/38

*Perfect classification of exemplars in the two categories has an AUC of 1 and a F1 of 1. AUC stands for Area Under the Curve in ROC analysis and F1. In order to compare AUC with the best known effect size measure Cohen's d, is included. Classifiers were run with default parameters of Weka and therefore without any parameter tuning*.

### Classification Accuracy Between Patients With Fibromyalgia and Healthy Controls With Preliminary Selection of Most Significant Attributes

In order to identify the most informative features based on the domains and subdomains administered, we run the classifiers using as input the best informative attributes.

In this dataset, classification accuracy was increased by a preliminary selection of the most informative input features (38). In Table 4 are displayed the accuracies achieved by 5 different classifiers with the preliminary best attributes selection.

**Table 4:** Accuracies as measured by % correct, AUC, and F1 obtained by five different ML classifiers (using 10-fold cross-validation and 5-fold cross-validation) with a preliminary best attributes selection.

**Classifier**	**Accuracy (%)**	**AUC**	**F1**	**Correct classification**	
**10-FOLD CROSS-VALIDATION**					
Naïve Bayes	86.48	0.90 (d = 1.81)	0.87	FMS 33/38	HC 33/38
Logistic regression	84.21	0.77 (d = 1.04)	0.84	FMS 32/33	HC 32/33
Simple logistics	94.74	0.93 (d = 2.09)	0.95	FMS 36/38	HC 36/38
Support vector machine	90.79	0.91 (d = 1.90)	0.91	FMS 34/35	HC 35/38
Random forest	88.16	0.93 (d = 2.09)	0.88	FMS 34/38	HC 33/38
**5-FOLD CROSS CROSS-VALIDATION**					
Naïve Bayes	82.89	0.90 (d = 1.81)	0.83	FMS 32/38	HC 31/38
Logistic regression	75.00	0.74 (d = 0.90)	0.75	FMS 30/38	HC 27/38
Simple logistics	86.84	0.87 (d = 1.59)	0.87	FMS 33/38	HC 33/38
Support vector machine	86.84	0.87 (d = 1.59)	0.87	FMS 33/38	HC 33/38
Random forest	85.53	0.92 (d = 1.98)	0.86	FMS 34/38	HC 31/38

*Perfect classification of exemplars in the two categories has an AUC of 1 and a F1 of 1. AUC stands for Area Under the Curve in ROC analysis and F1. In order to compare AUC with the best know effect size measure Cohen's d, is included. Classifiers were run with default parameters of Weka and therefore without any parameter tuning*.

In order to have insight of the best rule, based on the attributes selected classifier, J48 pruned three algorithm (using 10-fold cross validation), that correctly classify the subject in the two groups yielding an overall accuracy of 85.53%, the rule was the following:

*if TAS-20 (item 3) score is* ≤ *3:*

*and VAS score* ≤ *5, then the subject is classified as a control subject;*

*and VAS score* >*5, and TAS-20 (item 13) score is* ≤ *1, then the subject is classified as a FMS patient;*

*and VAS score* >*5, and TAS-20 (item 13) score is* >*1, then the subject is classified as a control subject*.

and

*if TAS-20 (item 3) score is* > *3:*

*and the gender is* =*F, then the subject is classified as a FMS patient;*

*and the gender is* =*M, then the subject is classified as control subject*.

Note that this is a rule which has different separated nested single and composed rules. The reported decision rule results in high accuracy in classifying FMS (33/38; accuracy = 87%) and HC subjects (32/38; accuracy = 84%), AUC = 0.82 (d = 1.29) and F1 = 0.85.

### Analysis Using Only TAS-20 Subscales and VAS Score as Predictors of Two Classes

When considering the previous tests and subscales scores, the single most efficient rule was obtained by J48 classifier (using 10-fold cross validation) that correctly classify the subjects in the two groups (FMS and HC) yielding an overall accuracy of 85.53%, where the rule highlighted the most sensitive scales were TAS-20 and VAS. In order to predict which of the TAS-20 subscales and cut-offs and VAS scale cut-off may be used as the single best predictor, we have considered the following subscales and features: TAS-20 (1–20 items), VAS total score, gender, and age. For this analysis, we have removed the gender, given that the sample was unbalance for variable (61 females, 15 males). The single most efficient rule was obtained by J48 algorithm, that correctly classifies the subjects in the two groups, yielded an overall accuracy of 88.16% (AUC = 0.88; d = 1.66; F1 = 0.88) and was the following:

***if TAS-20 (item 3) score is* ≤* 3:***

*and VAS score* ≤ *5, then the subject is classified as a control subject;*

*and VAS score* >*5, then the subject is classified as a FMS patient;*

and

***if TAS-20 (item 3) score is* >* 3:***

*and TAS-20 (item 7) is* ≤ *1, then the subject is classified as a control subject;*

*and TAS-20 (item 7) is* >*1, then the subject is classified as a FMS patient*.

### The Most Efficient TAS-20 Subset of Items Which Better Correlate With Fibromyalgia

In order to identify the most efficient items of the TAS-20 scale, we isolated the most efficient subset of items of the same scale through the algorithm developed by Hall (38), which identified those features highly correlated with the group and lower correlated among themselves. The TAS-20 subset items which better correlate with Fibromyalgia through attributes selection (10-fold cross-validation, stratified) were the following items: 1, 3, 7, 13, 14, and 15.

## Discussion and Conclusions

As far as we know, this is the first time that ML methods have been applied to fibromyalgia and related psychological domains. ML models have shown promising advantages in solving classification problems. The issue we intended to address using ML models was the following: which are the predictors that best distinguish between patients with FMS and healthy controls? Currently, clinical and research efforts have led to increasingly sophisticated methods to characterize and detect subthreshold alexithymia in patient with fibromyalgia, nevertheless there are still significant theoretical and practical challenges. Conventional statistical analysis doesn't allow to identify decision roles with a powerful translational effect in the clinical setting. The employment of the ML techniques allow to identify the best principles compare to the inferential statistic, which are both diagnostic rules and the accuracy. Models easily interpretable as those based on decision principles have allowed to identify decisional roles with a slightly reduction of accuracy. These decisional principles use the tests scores to detect the best predictors and rules useful by clinicians in the clinical practice.

Our study support the hypothesis that machine learning increases diagnosticity in psychometric evaluation of alexithymia in fibromyalgic patients.

The traditional statistical analysis has shown that all variables and scales considered have significant effect, ranging from small effect sizes to large effect, according to Cohen's interpretation (37). On the contrary classifications based on ML techniques reached higher accuracies, in the range of 84.21–88.16% with opaque classifiers and were able to (i) correctly classifies the subjects in the two groups (FMS and HC); (ii) to identified the subset of items which better correlate with the FMS group.

Our findings suggest that machine learning models analysis based on the TAS-20 subset of items scores accurately distinguish patients with fibromyalgia from healthy controls. The single most efficient rule, using only TAS subscales and VAS score as predictors of two classes, was obtained by J48 algorithm, that correctly classifies the subjects in the two groups, yielded an overall accuracy of 88.16% (AUC = 0.88; d = 1.66; F1 = 0.88).

The present research found that the vast majority of individual TAS-20 items (i.e., item 1, 3, 7, 13, 14, 15) that better correlated with fibromyalgia fell into the “Difficulty Identifying Feelings” feelings subscale of the alexithymia construct. These results may highlight the presence of a greater difficulty in identifying and expressing emotions and externally oriented thinking in patients with FMS compared to general population. Alexithymia as an emotional dysregulation trait, could play an important role in FM syndrome where individuals are less capable of adequately identifying physical sensations (i.e., somatic manifestations of emotions) leading them to misinterpret their emotional arousal as signs of disease (41, 42). This could amplify the perception of disease, bringing patients to seek medical care for symptoms for which there is no medical explanation.

Future replications in medico-clinical settings using participants with fibromyalgia are needed in order to corroborate our results.

## Data Availability Statement

The raw data supporting the conclusions of this article will be made available by the authors, without undue reservation, to any qualified researcher.

## Ethics Statement

The studies involving human participants were reviewed and approved by the Hospital of Pisa Ethics Committee and was conducted in accordance with the Declaration of Helsinki. All participants gave their written informed consent before participating in the study. The patients/participants provided their written informed consent to participate in this study.

## Author Contributions

CC and LB conceived the assessment. RC contributed to the data acquisition. GO contributed to the data analysis and data interpretation. GO, CC, LB, and AG drafted the manuscript. RC implemented the introduction associated with fibromyalgia and alexhitymia. All authors revised the manuscript and approved the final version to be published.

### Conflict of Interest

The authors declare that the research was conducted in the absence of any commercial or financial relationships that could be construed as a potential conflict of interest.
